# Supplementary material for: Modeling glioblastoma heterogeneity as a dynamic network of cell states
Source: Mol Syst Biol. 2021 Sep 16;17(9):e10105. doi: 10.15252/msb.202010105 (PMC8444284; doi:10.15252/msb.202010105)
Supplement: Supplementary file 6 — Source Data for Figure 5 [file MSB-17-e10105-s004.zip › Figure5A_sourcedata/GSEA_3071/nosigdb_stateI.GseaPreranked.1622795145664/gsea_report_for_na_pos_1622795145664.html]

Report for na\_pos 1622795145664 [GSEA]

| GS  follow link to MSigDB | GS DETAILS | SIZE | ES | NES | NOM p-val | FDR q-val | FWER p-val | RANK AT MAX | LEADING EDGE || 1 | OLSEN\_MESENCHYMAL | Details ... | 72 | 0.76 | 4.17 | 0.000 | 0.000 | 0.000 | 320 | tags=68%, list=9%, signal=73% |
| 2 | IVY\_CTMVP | Details ... | 79 | 0.71 | 4.09 | 0.000 | 0.000 | 0.000 | 508 | tags=71%, list=14%, signal=81% |
| 3 | NB\_KI\_CLUSTER18 | Details ... | 70 | 0.69 | 3.85 | 0.000 | 0.000 | 0.000 | 417 | tags=61%, list=12%, signal=68% |
| 4 | ANASTASSIOU\_MULTICANCER\_INVASIVENESS\_PMID\_22208948 | Details ... | 39 | 0.82 | 3.83 | 0.000 | 0.000 | 0.000 | 284 | tags=74%, list=8%, signal=80% |
| 5 | DOROSZKO\_U3054MG\_CLUSTER\_2 | Details ... | 227 | 0.54 | 3.70 | 0.000 | 0.000 | 0.000 | 358 | tags=46%, list=10%, signal=48% |
| 6 | VANGRONINGEN\_MES | Details ... | 269 | 0.51 | 3.53 | 0.000 | 0.000 | 0.000 | 800 | tags=59%, list=23%, signal=71% |
| 7 | RICHARDS\_VERHAAK\_CANCERCELL\_2010\_MESENCHYMAL | Details ... | 74 | 0.62 | 3.41 | 0.000 | 0.000 | 0.000 | 874 | tags=78%, list=25%, signal=102% |
| 8 | GANDAL\_IBD\_UP\_PMID29439242 | Details ... | 56 | 0.63 | 3.29 | 0.000 | 0.000 | 0.000 | 275 | tags=46%, list=8%, signal=50% |
| 9 | RICHARDS\_NOWAKOWSKI\_SCIENCE\_2017\_MURAL\_UPREG | Details ... | 354 | 0.45 | 3.28 | 0.000 | 0.000 | 0.000 | 749 | tags=49%, list=21%, signal=56% |
| 10 | SUVA\_MES1\_PMID\_31327527 | Details ... | 30 | 0.69 | 3.06 | 0.000 | 0.000 | 0.000 | 275 | tags=53%, list=8%, signal=57% |
| 11 | RICHARDS\_NEFTEL\_CELL\_2019\_MES1 | Details ... | 30 | 0.69 | 3.02 | 0.000 | 0.000 | 0.000 | 275 | tags=53%, list=8%, signal=57% |
| 12 | WANG\_MESENCHYMAL\_PMID\_28697342 | Details ... | 24 | 0.71 | 2.90 | 0.000 | 0.000 | 0.000 | 451 | tags=58%, list=13%, signal=66% |
| 13 | DALMO\_EXTINCTION\_UNPUB | Details ... | 24 | 0.71 | 2.89 | 0.000 | 0.000 | 0.000 | 451 | tags=58%, list=13%, signal=66% |
| 14 | GANDAL\_AUTISM\_UP\_PMID29439242 | Details ... | 56 | 0.55 | 2.84 | 0.000 | 0.000 | 0.000 | 674 | tags=63%, list=19%, signal=76% |
| 15 | SEGERMAN\_MULTITHERAPY\_RESISTANCE\_FIG5A\_PMID\_27974212 | Details ... | 24 | 0.67 | 2.70 | 0.000 | 0.000 | 0.000 | 918 | tags=83%, list=26%, signal=112% |
| 16 | NB\_KI\_CLUSTER17 | Details ... | 93 | 0.45 | 2.65 | 0.000 | 0.000 | 0.000 | 800 | tags=60%, list=23%, signal=76% |
| 17 | NB\_KI\_CLUSTER11 | Details ... | 40 | 0.52 | 2.54 | 0.000 | 0.000 | 0.000 | 872 | tags=68%, list=25%, signal=89% |
| 18 | NB\_KI\_CLUSTER13 | Details ... | 79 | 0.43 | 2.44 | 0.000 | 0.000 | 0.000 | 865 | tags=58%, list=25%, signal=76% |
| 19 | RICHARDS\_ZHONG\_NATURE\_2018\_MICROGLIA\_UPREG | Details ... | 135 | 0.36 | 2.22 | 0.000 | 0.000 | 0.009 | 1071 | tags=60%, list=30%, signal=83% |
| 20 | DOROSZKO\_U3013MG\_CLUSTER\_1 | Details ... | 33 | 0.49 | 2.21 | 0.003 | 0.001 | 0.012 | 236 | tags=30%, list=7%, signal=32% |
| 21 | SINGH\_SOX2\_DOWNREGULATED\_TRANSCRIPTS\_PMID\_28122245 |  | 59 | 0.41 | 2.19 | 0.000 | 0.001 | 0.014 | 442 | tags=36%, list=13%, signal=40% |
| 22 | RICHARDS\_NOWAKOWSKI\_SCIENCE\_2017\_CHOROID\_UPREG |  | 238 | 0.31 | 2.17 | 0.000 | 0.001 | 0.015 | 813 | tags=43%, list=23%, signal=52% |
| 23 | KRONA\_ROSEN\_WHITE\_MATTER\_INVASION |  | 144 | 0.32 | 2.09 | 0.000 | 0.001 | 0.035 | 421 | tags=31%, list=12%, signal=34% |
| 24 | RICHARDS\_NOWAKOWSKI\_SCIENCE\_2017\_MICROGLIA\_UPREG |  | 312 | 0.29 | 2.08 | 0.000 | 0.001 | 0.037 | 798 | tags=41%, list=23%, signal=48% |
| 25 | SUVA\_MES2\_PMID\_31327527 |  | 27 | 0.46 | 2.00 | 0.000 | 0.002 | 0.065 | 799 | tags=67%, list=23%, signal=86% |
| 26 | RICHARDS\_NEFTEL\_CELL\_2019\_MES2 |  | 27 | 0.46 | 1.96 | 0.003 | 0.003 | 0.084 | 799 | tags=67%, list=23%, signal=86% |
| 27 | OLSEN\_SCPLIKE |  | 64 | 0.34 | 1.83 | 0.000 | 0.008 | 0.222 | 427 | tags=41%, list=12%, signal=45% |
| 28 | ALMSTEDT\_VGR\_DIFF |  | 442 | 0.24 | 1.80 | 0.000 | 0.009 | 0.246 | 505 | tags=27%, list=14%, signal=27% |
| 29 | ROSEN\_KRONA\_TI\_UNPUBL |  | 30 | 0.40 | 1.72 | 0.026 | 0.017 | 0.412 | 312 | tags=30%, list=9%, signal=33% |
| 30 | NB\_KI\_CLUSTER14 |  | 105 | 0.28 | 1.69 | 0.003 | 0.019 | 0.470 | 811 | tags=40%, list=23%, signal=50% |
| 31 | NB\_KI\_CLUSTER23 |  | 132 | 0.26 | 1.63 | 0.004 | 0.026 | 0.594 | 992 | tags=53%, list=28%, signal=71% |
| 32 | DOROSZKO\_U3180MG\_CLUSTER\_6 |  | 27 | 0.38 | 1.57 | 0.035 | 0.038 | 0.738 | 800 | tags=59%, list=23%, signal=76% |
| 33 | RICHARDS\_CAHOY\_JNEUROSCI\_2014\_MOG\_POS |  | 261 | 0.22 | 1.53 | 0.000 | 0.048 | 0.817 | 622 | tags=28%, list=18%, signal=31% |
| 34 | IVY\_CTPAN |  | 70 | 0.28 | 1.52 | 0.026 | 0.048 | 0.826 | 749 | tags=41%, list=21%, signal=52% |
| 35 | GANDAL\_ALCOHOLADDICTION\_UP\_PMID29439242 |  | 51 | 0.28 | 1.43 | 0.056 | 0.080 | 0.947 | 478 | tags=31%, list=14%, signal=36% |
| 36 | NB\_KI\_CLUSTER20 |  | 16 | 0.39 | 1.40 | 0.096 | 0.093 | 0.975 | 898 | tags=63%, list=26%, signal=84% |
| 37 | KRONA\_ROSEN\_PERIVASCULAR\_INVASION |  | 36 | 0.29 | 1.36 | 0.118 | 0.118 | 0.991 | 882 | tags=44%, list=25%, signal=59% |
| 38 | NB\_KI\_CLUSTER15 |  | 60 | 0.25 | 1.32 | 0.099 | 0.140 | 0.995 | 978 | tags=52%, list=28%, signal=70% |
| 39 | RICHARDS\_VERHAAK\_CANCERCELL\_2010\_NEURAL |  | 23 | 0.31 | 1.30 | 0.164 | 0.155 | 0.998 | 1261 | tags=61%, list=36%, signal=94% |
| 40 | RICHARDS\_LIDDELOW\_NATURE\_2017\_A1\_REACTIVE\_ASTROCYTES |  | 21 | 0.32 | 1.28 | 0.183 | 0.169 | 1.000 | 695 | tags=48%, list=20%, signal=59% |
| 41 | ALLEN\_115\_OLIGOL4\_6MOBPCOL18A1 |  | 16 | 0.34 | 1.25 | 0.218 | 0.186 | 1.000 | 76 | tags=19%, list=2%, signal=19% |
| 42 | DOROSZKO\_U3054MG\_CLUSTER\_3 |  | 95 | 0.21 | 1.24 | 0.141 | 0.195 | 1.000 | 456 | tags=23%, list=13%, signal=26% |
| 43 | NB\_KI\_CLUSTER16 |  | 54 | 0.23 | 1.15 | 0.279 | 0.294 | 1.000 | 731 | tags=41%, list=21%, signal=51% |
| 44 | NB\_KI\_CLUSTER12 |  | 34 | 0.23 | 1.04 | 0.391 | 0.468 | 1.000 | 542 | tags=26%, list=15%, signal=31% |
| 45 | RICHARDS\_CAHOY\_JNEUROSCI\_2013\_OL\_MYEL |  | 414 | 0.14 | 1.01 | 0.472 | 0.527 | 1.000 | 627 | tags=21%, list=18%, signal=22% |
| 46 | DOROSZKO\_U3054MG\_CLUSTER\_1 |  | 16 | 0.27 | 1.00 | 0.463 | 0.530 | 1.000 | 752 | tags=50%, list=21%, signal=63% |
| 47 | ALMSTEDT\_17Q\_MSIGDB |  | 114 | 0.16 | 0.99 | 0.532 | 0.538 | 1.000 | 668 | tags=25%, list=19%, signal=29% |
| 48 | GANDAL\_SCHIZOPHRENIA\_DN\_PMID29439242 |  | 16 | 0.26 | 0.94 | 0.515 | 0.624 | 1.000 | 832 | tags=50%, list=24%, signal=65% |
| 49 | RICHARDS\_NOWAKOWSKI\_SCIENCE\_2017\_GLYC\_UPREG |  | 269 | 0.13 | 0.89 | 0.798 | 0.699 | 1.000 | 1131 | tags=44%, list=32%, signal=60% |
| 50 | GANDAL\_DEPRESSION\_DN\_PMID29439242 |  | 18 | 0.20 | 0.75 | 0.758 | 0.880 | 1.000 | 950 | tags=44%, list=27%, signal=61% |
| 51 | DOROSZKO\_U3054MG\_CLUSTER\_9 |  | 166 | 0.10 | 0.65 | 0.992 | 0.953 | 1.000 | 750 | tags=22%, list=21%, signal=27% |
| 52 | NB\_KI\_CLUSTER8 |  | 93 | 0.09 | 0.53 | 0.997 | 0.981 | 1.000 | 562 | tags=16%, list=16%, signal=19% |
Table: Gene sets enriched in phenotype **na **[plain text format]****
